# Episodic Positive Selection in the Evolution of Avian Toll-Like Receptor Innate Immunity Genes

**DOI:** 10.1371/journal.pone.0089632

**Published:** 2014-03-03

**Authors:** Catherine E. Grueber, Graham P. Wallis, Ian G. Jamieson

**Affiliations:** 1 Allan Wilson Centre for Molecular Ecology and Evolution, Dunedin, New Zealand; 2 Department of Zoology, University of Otago, Dunedin, New Zealand; University of Uppsala, Sweden

## Abstract

Toll-like receptors (TLRs) are a family of conserved pattern-recognition molecules responsible for initiating innate and acquired immune responses. Because they play a key role in host defence, these genes have received increasing interest in the evolutionary and population genetics literature, as their variation represents a potential target of adaptive evolution. However, the role of pathogen-mediated selection (i.e. episodic positive selection) in the evolution of these genes remains poorly known and has not been examined outside of mammals. A recent increase in the number of bird species for which TLR sequences are available has enabled us to examine the selective processes that have influenced evolution of the 10 known avian TLR genes. Specifically, we tested for episodic positive selection to identify codons that experience purifying selection for the majority of their evolution, interspersed with bursts of positive selection that may occur only in restricted lineages. We included up to 23 species per gene (mean = 16.0) and observed that, although purifying selection was evident, an average of 4.5% of codons experienced episodic positive selection across all loci. For four genes in which sequence coverage traversed both the extracellular leucine-rich repeat region (LRR) and transmembrane/intracellular domains of the proteins, increased positive selection was observed at the extracellular domain, consistent with theoretical predictions. Our results provide evidence that episodic positive selection has played an important role in the evolution of most avian TLRs, consistent with the role of these loci in pathogen recognition and a mechanism of host-pathogen coevolution.

## Introduction

Immune genes are ideal for studying how evolutionary mechanisms influence the genetic diversity of natural populations, as such loci experience a variety of selection pressures from a wide diversity of coevolving pathogens with which animals are associated [1], [2]. While genes of the major-histocompatibility complex (MHC) have been widely used for assaying levels of functional diversity, variation at other immunity loci also underlies variation in individual immune response, and is thus of interest in evolutionary and population genetics [3]–[6]. Even from a conservation genetics perspective, assaying variation at functional genomic regions provides a better understanding of how processes such as population bottlenecks and inbreeding can impact the adaptive potential of threatened species [7]. However, the role of selection in shaping diversity of other (non-MHC) aspects of the immune system, such as innate immunity, remains poorly understood in natural populations [7]–[9].

One important family of innate-immunity genes is the toll-like receptors (TLRs): a group of genes that recognise a wide diversity of pathogens and are responsible for initiating both the innate and acquired immune responses through binding of pathogen-associated molecular patterns (PAMPs) [10]–[12]. This crucial role was discovered as recently as 1997 [12], and since then genes assigned to the TLR family have been characterised in many diverse animal groups (e.g. [13]). Recent evidence suggests that changes to both the TLR repertoire and its gene sequences occur relatively rapidly over evolutionary time [14], likely a result of the varying pathogen pressures experienced by different taxa (e.g. [15]).

Previous studies of TLR evolution have found a high degree of purifying selection [15]–[18], some evidence of balancing selection [19], and a small number of studies have found residues under positive selection in several genes [9], [17], [18]. However, it is likely that TLRs also experience spatiotemporal variation in selection, as a result of Red-Queen type selection pressure from co-evolving pathogens. For example, under episodic selection, many codons experience purifying selection for the majority of their evolution, with bursts of strong positive selection within particular lineages [20]. Mutations at such sites may experience transient positive selection, followed by purifying selection to maintain the change, and likely play a key role in adaptive evolution [20]–[22]. To date, the extent to which TLR diversity is impacted by pathogen-mediated selection (i.e. episodic positive selection) has been examined in just one vertebrate subfamily, the Murinae (rats and mice), and for only two genes (*TLR4* and *TLR7* [8]; 10 TLR genes are known in many mammals [18]). Although this study found episodic selection at a number of sites involved with pathogen recognition in *TLR4* (but not *TLR7* [18]), the role of this type of selection in other vertebrate taxa, or on other genes, remains unknown.

A previous study examined pervasive positive selection on avian TLRs [17], and since then the number of bird species for which TLR sequences are available has doubled (e.g. [23], see also Results); we use this much larger dataset to test for episodic positive selection. We chose to examine birds because two recent studies have reported high levels of TLR haplotype diversity in wild bird populations [7], [17], and a further analysis suggested that genotypic variation can influence variation in juvenile survival in a wild population of native New Zealand robins [24]. We therefore predict that episodic positive selection has played an important role in the evolution of these genes, consistent with a pathogen-mediated co-evolutionary regime.

The avian TLR repertoire, first characterised following completion of the chicken genome [13], has a number of characteristics that differentiate it from that of other vertebrates, including mammals. Although four avian TLRs have clear orthologs in other vertebrates (*TLR3*, *TLR4*, *TRL5* and *TLR7*), *TLR7* appears to be duplicated in members of the order Passeriformes [7], [17], [25]. In addition, *TLR2A* and *TLR2B* arose by duplication of vertebrate *TLR2* early in avian evolution, while the *TLR1LA* (“*TLR1*-like”) and *TLR1LB* gene-duplicates belong to the vertebrate *TLR1/6/10* superfamily [13]. *TLR21* is shared with bony fishes and *Xenopus* [13]. *TLR15* appears to be unique to birds and reptiles [13], [26]. Together these genes produce proteins that recognise a diversity of PAMPs [27], such as lipopolysaccharide of Gram-negative bacteria (*TLR4* [28]), yeast-derived compounds (*TLR15* [26]), or CpG motifs of viral DNA (*TLR21* [29]).

In addition to examining episodic selection in avian TLRs, we test the hypothesis that greater evidence for positive selection should occur in the extracellular domain characterised by leucine-rich repeats (LRRs) than at a signal/transmembrane domain or an intracellular toll/interleukin I resistance (TIR) domain [18], [30], [31]. The extracellular domain, responsible for PAMP binding, is thought to show greater variation than the TIR domain, which is highly conserved to maintain integrity of intracellular signalling cascades that initiate immune responses [18], [31]. Thus, the different domains of TLR molecules likely evolve under different selection regimes, a prediction that has been upheld in a small number of mammal studies [8], [18], [31], but never tested in other taxa such as birds.

## Materials and Methods

To test our hypotheses, we retrieved avian TLR sequences from Genbank (Table S1). Sequences were initially aligned using Geneious v5.5 [32], and the alignment refined using Clustalx v2.1 [33] and MEGA v5.1 [34]. Sequences varied in length, so we trimmed back our alignments to include only codons covered by >50% of sampled species; we excluded species with short sequences that covered <50% of the overall alignment. Because *TLR7* appears to be duplicated in passerines [7], [17], [25], results for this locus should be interpreted with caution, as passerine sequences may represent co-amplification of paralogous loci. We attempted to minimise this issue by using haplotype data from passerine *TLR7*-type 1 where available (e.g. cloned sequences).

### Evolutionary Analyses

To facilitate comparison of our results to previous work, we tested for evidence of positive selection using the single likelihood ancestor counting (SLAC) and random-effects likelihood (REL) methods, as well as making the first examination of episodic selection in avian TLRs using the mixed-effects model of evolution (MEME). Phylogenetic relationships among sequences for each TLR locus were estimated using the neighbour-joining method implemented in the HyPhy package [35] available on the Datamonkey webserver (http://www.datamonkey.org, accessed October 2013 [36]); the resultant phylogenetic trees were visualised using functions available in the package “ape” [37] for R [38] (Figure S1). These trees were then used as input for phylogenetic tests of positive selection at each gene using the HyPhy package. For each gene, we used the model selection tool [39] to determine the most appropriate nucleotide substitution model to use.

The SLAC model reconstructs ancestral sequences and then uses counts of *d_S_* and *d_N_* at each codon position; the REL model uses the observed substitution rates, in comparison to a fitted rate distribution across sites (Kosakovsky Pond and Frost 2005). SLAC and REL both have limitations relative to other likelihood methods in that SLAC lacks power in certain datasets, while REL can be susceptible to false positives; therefore the use of appropriate significance thresholds and basing interpretations on consensus outcomes is generally considered to be a reasonable approach [40]. We used α = 0.1 for SLAC and a Bayes factor >50 for REL, as our significance thresholds.

MEME is a generalisation of the fixed-effects likelihood (FEL) approach, where ω at each codon is free to vary across lineages, thereby identifying residues that have undergone episodic selection (i.e. positive selection that varies temporally throughout the tree) [20]. MEME shows superior performance over FEL for a variety of tree topologies and selection scenarios and is expected to show greater power over site models (such as SLAC and REL) [20]. Where selection is pervasive (not temporally varying), MEME shows similar power to other approaches [20]. Maximum-likelihood branch-site methods are robust to high levels of divergence among taxa [41]; divergences we report for our alignments are similar to those of the alignments upon which MEME was initially tested [20]. We used the default α = 0.1 as our significance threshold for MEME; use of a more stringent threshold (α = 0.05) resulted in fewer statistically significant sites, but did not change our overall conclusions regarding the localisation of positively selected codons (see below).

Because MEME allows the mapping of positively selected sites to branches in a phylogeny, we were able to identify the proportion of positively selected sites that occurred on internal versus terminal branches, and compare this value to a null-hypothesis of uniform distribution. To compare the locations of positively selected sites on each phylogeny, calculations of internal and terminal branch lengths were performed using functions available in the R-package “ape”. Physicochemical distances between inferred amino acid substitutions, or among amino acid states at variable sites, were quantified using Grantham's [42] distance matrix. For each locus, we used a Student's *t*-test to compare the divergences of all inferred substitutions across the alignment, to the mean distances among the amino acids observed at each positively selected site. We used a null hypothesis of equal means (two-sided test).

### Localisation of positively selected codons

We assessed the functional significance of positively selected codons by examining the distribution of these results across functional domains of each gene, as delineated using the software LRRfinder [43], and using chicken protein sequences as the reference (*TLR1LA*: BAD67422.1, *TLR1LB*: ABF67957.1, *TLR2A*: NP_989609.1, *TLR2B*: BAB16842.1, *TLR3*: NP_001011691.3, *TLR4*: AAL49971.1, *TLR5*: CAF25167.1, *TLR7*: NP_001011688.1, *TLR15*: NP_001032924.1, *TLR21*: NP_001025729.1). For the four genes for which it was possible to compare selection in LRR and other domains of the gene (*TLR1LA*, *TLR1LB*, *TLR2A*, *TLR2B*), we examined whether the mean normalised *d_N_*-*d_S_* values (as determined by the SLAC method) differed between domain types (LRRs and “other”), using a linear model for each gene. In each model, the codon domain (LRR/other) was a categorical predictor variable and the *d_N_*-*d_S_* value for each codon was the response variable.

We also tested whether statistically significant findings of selection were disproportionately distributed in LRR or in other domains of the each gene. We used a two-sided Fisher's exact test where the null expectation was that significant findings would be uniformly distributed across LRR and “other” codons. Our observed value was the proportion of significant codons that occurred within LRR domains, from the total number of significant codons detected. We visualised these comparisons with binomial 95% confidence intervals for the observed values, obtained using the Agresti-Coull method [44] where *N*≥10 or the exact method for datasets with smaller sample sizes. Both confidence interval methods are implemented in the package “binom” [45] for R.

Crystal structures were available for the mammalian homologs of two of the loci studied here (*TLR3* and *TLR4*). To facilitate localisation of positively selected sites on the crystal structures of *TLR3* and *TLR4*, we first generated alignments of chicken TLR proteins against each of the mammalian products (mouse *TLR3*, MMDB ID 64341 and human *TLR4*, MMDB ID 70004) using Geneious (Figure S2 and S3). We mapped the location of positively selected codons (as identified in the MEME analysis) onto the three-dimensional mammalian protein structure using the NCBI application Cn3D [46]. Note that indels were introduced to improve homology of both alignments (Figure S2 and S3); we thus consider the codon mappings to be approximate. Unless otherwise noted, all statistical analyses performed herein were conducted using functions available in either the “base” or “stats” packages of R.

## Results

Our alignments contained between 9 and 23 taxa (mean = 16.0, Table 1) including an additional 7.6 species per gene (range 2 [*TLR2A*] to 13 [*TLR7*]) compared to the earlier analysis of avian TLR evolution [17]. Our data incorporate two additional avian orders, the Apterygiformes (kiwi) (*Apteryx mantelli* in *TLR1LA*, *TLR2B*, *TLR3*, *TLR5*, *TLR7*) and Gruiformes (crane-like birds) (*Porphyrio hochstetteri* in *TLR1LA*, *TLR1LB*, *TLR3*, *TLR4*, *TLR5*, *TLR7*, *TLR15*, *TLR21*) (Table S1).

**Table 1:** Selection and diversity statistics for ten avian TLR alignments.

Alignment			Diversity estimates			Sites under selection			
Locus	Taxa	bp (aa)	*d_N_* (SE)	*d_S_* (SE)	*d_N_*/*d_S_*	SLAC	REL	Either*	MEME
*TLR1LA*	19	1,161 (387)	0.098 (0.007)	0.260 (0.015)	0.346	1	5	5 (1.3%)	13 (3.4%)
*TLR1LB*	16	948 (316)	0.115 (0.009)	0.267 (0.018)	0.386	1	0	1 (0.3%)	14 (4.4%)
*TLR2A*	11	1,239 (413)	0.147 (0.011)	0.274 (0.017)	0.474	0	6	6 (1.5%)	24 (5.8%)
*TLR2B*	14	1,191 (397)	0.108 (0.008)	0.311 (0.019)	0.376	1	0	1 (0.3%)	12 (3.0%)
*TLR3*	17	1,125 (375)	0.056 (0.005)	0.210 (0.013)	0.309	3	9	9 (2.4%)	11 (2.9%)
*TLR4*	18	810 (270)	0.153 (0.013)	0.294 (0.020)	0.487	1	10	10 (3.7%)	19 (7.0%)
*TLR5*	19	1,251 (417)	0.107 (0.007)	0.214 (0.012)	0.551	6	8	10 (2.4%)	34 (8.2%)
*TLR7*	23	894 (298)	0.090 (0.008)	0.266 (0.016)	0.336	0	3	3 (1.0%)	13 (4.4%)
*TLR15*	14	1,320 (440)	0.182 (0.011)	0.470 (0.025)	0.435	2	5	5 (1.1%)	19 (4.3%)
*TLR21*	9	756 (252)	0.179 (0.016)	0.416 (0.029)	0.196	0	0	0 (0.0%)	3 (1.2%)
Mean	16.0	1,070 (357)	0.124	0.298	0.390	1.5	4.6	5.0 (1.4%)	16.2 (4.5%)

NOTE. **bp** base-pairs, **aa** amino acids, ***d_N_*** (number of non-synonymous substitutions per non-synonymous site) and ***d_S_*** (number of synonymous substitutions per synonymous site) were estimated using the modified Nei-Gojobori/Jukes-Cantor method [61], **SE** of *d_N_* and *d_S_* were estimated using 500 bootstrap iterations in MEGA, ***d_N_*** **/** ***d_S_*** was estimated using the SLAC method implemented in datamonkey,

* “Either” indicates the number (and percentage) of codons at which selection was detected with either the SLAC or REL methods.

**Figure 1:**
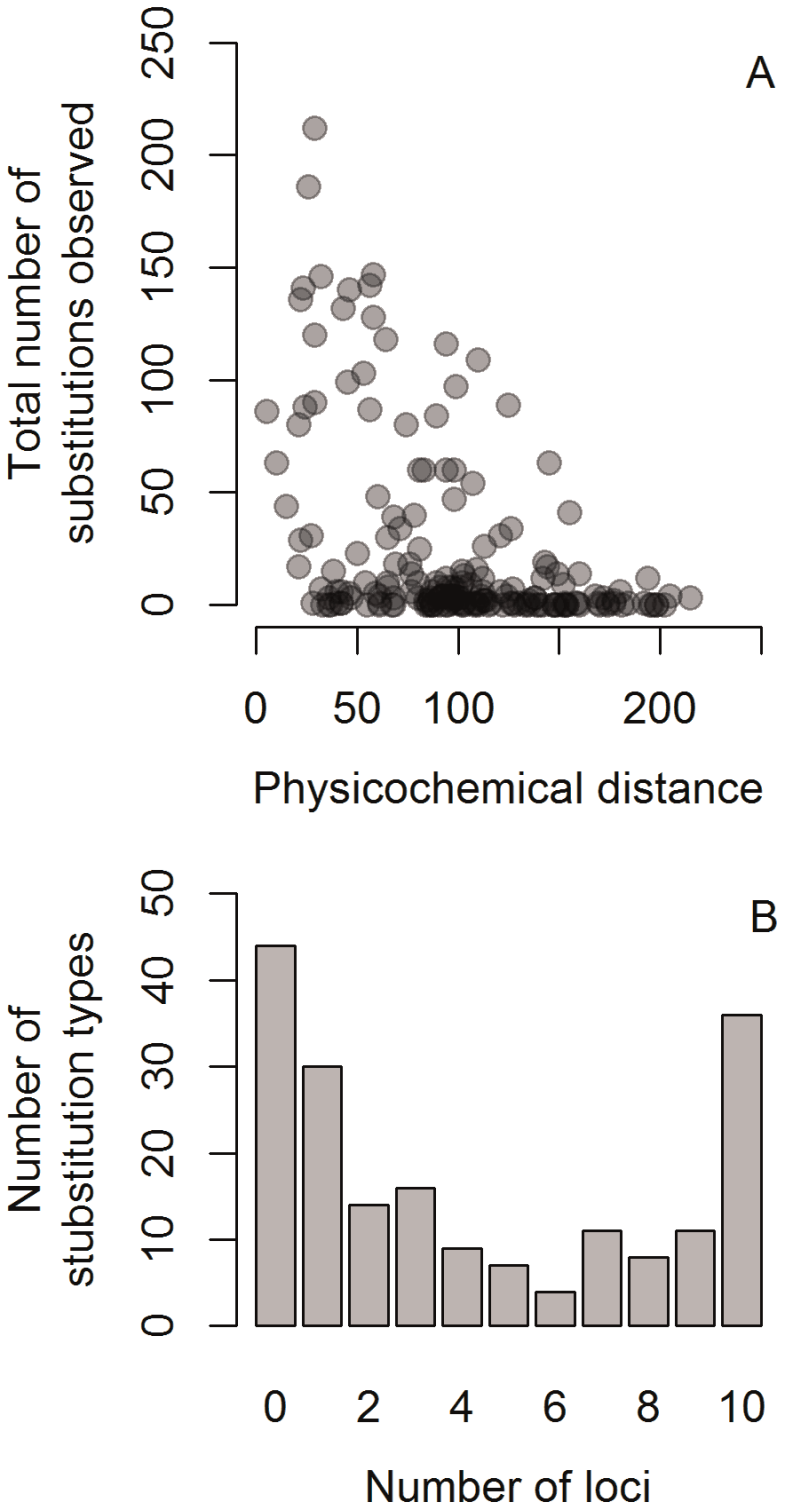
Characteristics of substitutions occurring in ten Toll-like receptor loci in birds. Frequency with which each amino-acid substitution type (*N* = 190 possible substitutions) was observed, relative to their predicted physicochemical distances [42] (A). The number of amino acid substitution types (*N* = 190) that were observed at least once in each of 10 avian TLR loci, as a function of the number of alignments in which each type was observed (B).

Across all genes, we detected 4,441 amino acid substitutions (excluding codons with a nucleotide mixture); the frequencies of each type (*N* = 190 possible amino-acid substitution types total) were inversely correlated with Grantham's [42] physicochemical distance matrix (Pearson correlation = −0.44, Figure 1A; counts of each substitution are shown in Figure S4). Of all 190 possible amino-acid substitution types, 36 (19%) were observed in the alignments of all 10 TLRs, while 44 (23%) were not observed at all (Figure 1B).

### Evolutionary analyses

Comparison of rates of synonymous and non-synonymous substitution revealed purifying selection for all genes (ω values were <1 for all loci, Table 1). Despite evidence of purifying selection acting on the proteins overall, positive selection (as detected using the SLAC, REL or MEME methods) was observed across all loci (Table 1), at a total of 171 sites (Table S2). These codons included 18 (46%) of the 39 sites previously identified as under positive selection in avian TLRs [17] (Table S2). The SLAC and REL methods together detected statistically significant positive selection at an average of 1.4% of codons (Table 1). By contrast, the MEME approach detected episodic positive selection at an average of over three times as many codons (mean = 4.5%; Table 1).

**Figure 2:**
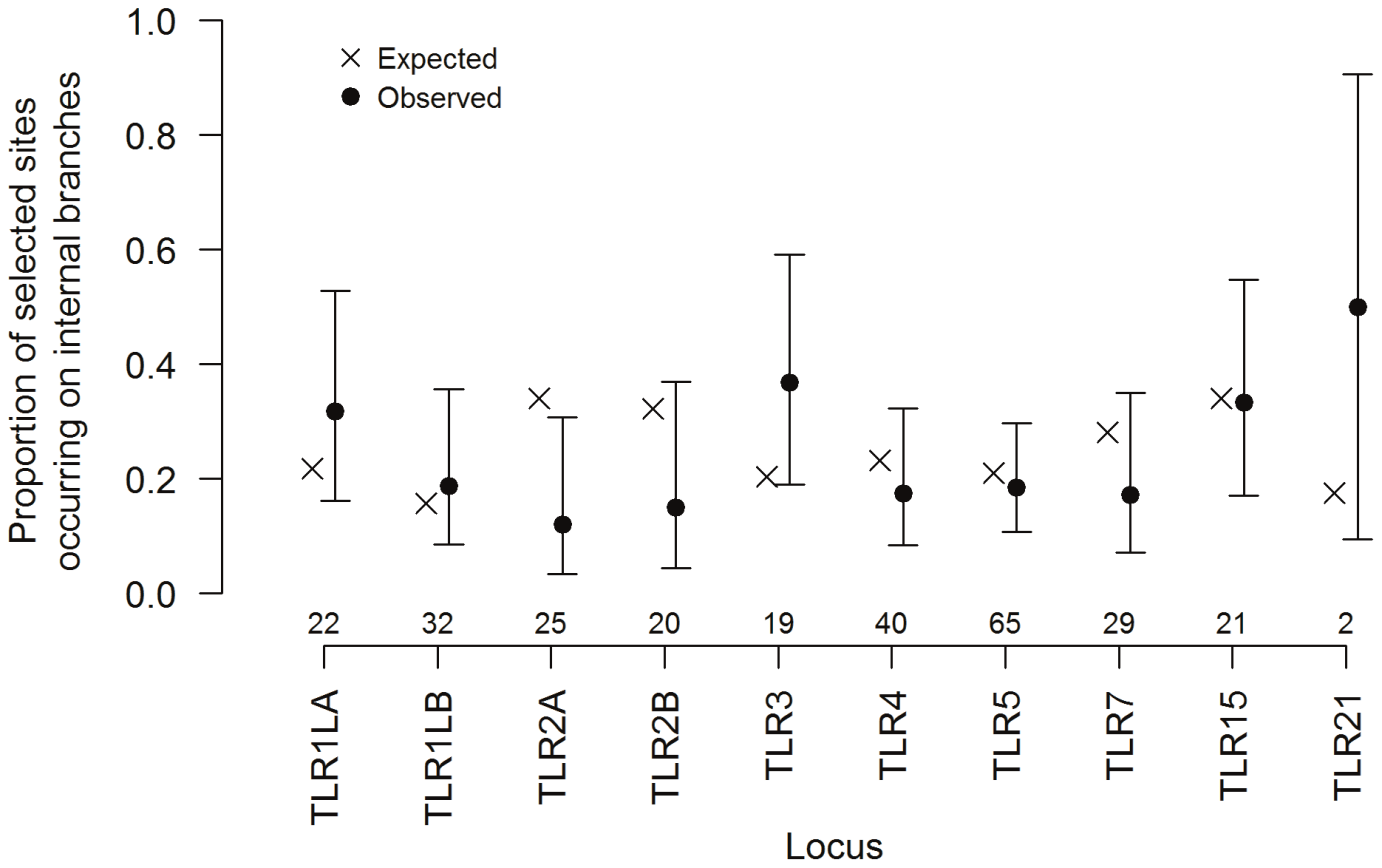
Proportion of positively selected sites detected on internal or terminal branches of each gene tree. Positively selected sites were determined by MEME analysis; trees are available in Figure S1. Points indicate observed proportions; error bars are Agresti-Coull binomial 95% confidence intervals [44] calculated using the R package “binom” [45]. Crosses indicate the proportion expected under a null hypothesis of random distribution, based on the sum of internal/terminal branch lengths. Values along the *x*-axis are sample sizes of mapped MEME results; note that these values can be larger than those presented in Table 1, as some sites are selected on multiple branches, while not all sites under selection were mapped to specific branches (i.e. they showed pervasive selection) (see also Figure S5).

We found that for most loci, the temporal distribution of positive selection throughout the phylogeny was consistent with a null model of uniform distribution, with one exception: *TLR2A* showed a significantly greater proportion of positively selected sites on terminal branches (i.e. species-specific selection) than expected (Figure 2), many of these in *Petroica* (Figure S5). The amino acid variants observed at positively selected sites showed slightly higher mean physicochemical divergence from each other than inferred substitutions generally (positively selected sites mean distance = 79.3; mean distance of all substitutions = 63.1; Figure S6); these differences were statistically significant at α = 0.05 for 8 of the 10 genes studied (test statistics provided in Table S3). Although sites with a high degree of variation may be more likely to be identified as positively selected in the first place, we note this high amino acid diversity was not essential to detecting positive selection, as statistically significant findings of selection were observed at sites with both few (i.e. 2) and many (up to 8) amino acid variants across the alignment (Table S2).

### Localisation of positively selected codons in TLR domains

**Figure 3:**
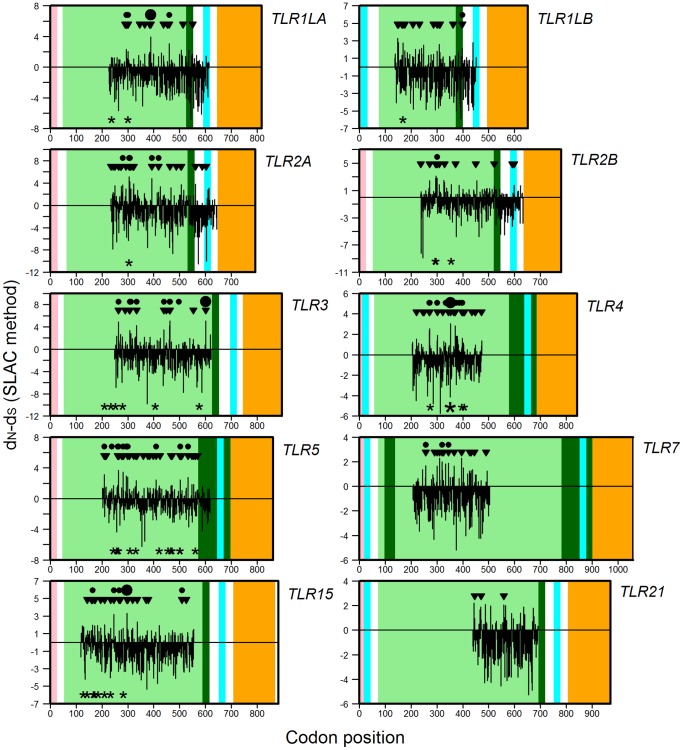
Map of nucleotide substitution patterns at Toll-like receptor loci in birds. The analysed region of each gene is positioned on an *x*-axis aligned with chicken TLRs. Within each panel of the figure, vertical black lines indicate the degree of positive (*d_N_*-*d_S_*>0) or negative (*d_N_*-*d_S_*<0) selection at each codon site. Note that the *y*-axes vary in scale. Panel backgrounds are shaded to illustrate locations of conserved domains within the alignment region (based on LRRfinder [43]) (pink = signal domain; light green = extracellular LRR regions; dark green = C-terminal LRR regions; cyan = transmembrane domain; orange = TIR). Within each panel, three rows of symbols indicate codons for which statistically significant evidence of selection was detected: the top row of symbols (circles) indicates codons under positive selection in the current study, using either the SLAC or REL methods, larger symbols are used where the two methods correspond. The second row of symbols (triangles) indicates codons detected in the current study as being under episodic diversifying selection, using the MEME analysis. The third row of symbols (asterisks) indicates sites previously observed to be under positive selection in a smaller avian alignment of the same regions [17] using either the SLAC or REL methods, larger symbols indicate codons in which the two methods correspond.

**Figure 4:**
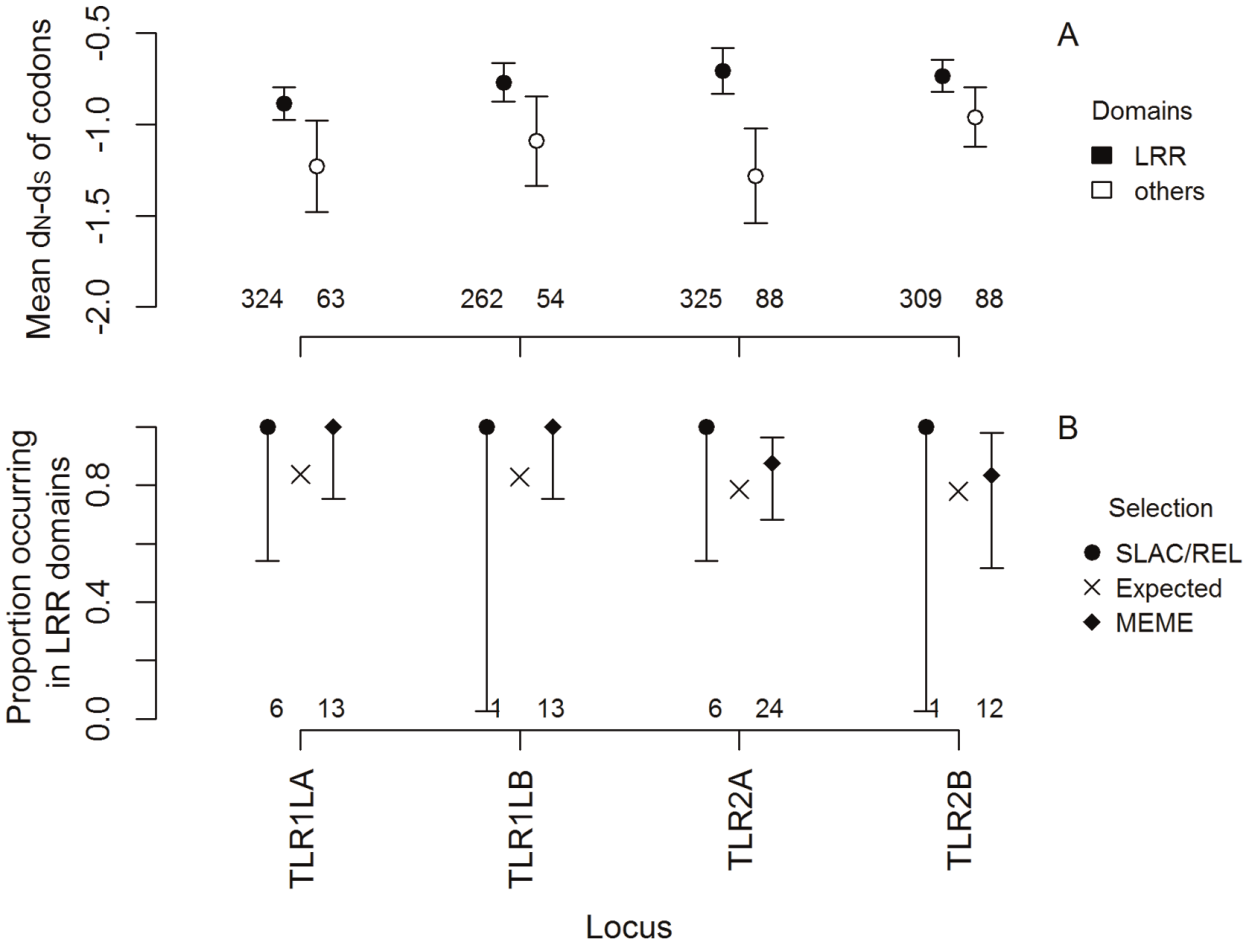
Differences in selection across LRR and other domains of four TLR genes in birds. In A, filled circles indicate the mean *d_N_*-*d_S_* values (estimated using the SLAC method) of codons located in LRR domains, while open circles indicate the mean values of all other codons; error bars indicate the standard error; numbers of codons assayed in each region are shown along *x*-axis. Panel B shows the proportion of statistically significant results for codons under positive selection (as determined by the SLAC or REL methods, circles on left, hits at the same site from multiple methods were counted separately) or episodic diversifying selection (as determined by the MEME method, diamonds on right), that fell within LRR domains of each toll-like receptor gene. Crosses indicate the proportion of results expected to fall within LRR domains under the null hypothesis, given the number of genotyped codons in those regions. Total numbers of significant findings by each method are shown along the *x*-axis; error bars are 95% confidence intervals for binomial proportions (see Methods).

Because our alignments do not traverse the entire coding sequence of each gene, comparisons between LRR and non-LRR domains of the genes were only possible for *TLR1LA*, *TLR1LB*, *TLR2A* and *TLR2B* (for the remaining five genes, each alignment included only the LRR domain). For these four genes, sequencing across LRR and non-LRR regions was uneven, with an average 81% of the data occurring in the LRR region (Figure 3). Nevertheless we observed that mean normalised *d_N_*-*d_S_* was generally higher in LRR domain than other domains, and for one gene (*TLR2A*) this trend was statistically significant at α = 0.05 (means and sample sizes shown in Figure 4A; full test statistics provided in Table S4). A greater number of statistically significant findings of positive selection were observed in LRR domains than expected under uniform distribution (Figure 4B), although these results did not differ significantly from our null expectations of a uniform distribution (Fisher's exact test, all *P*-values>0.1), probably as a result of the aforementioned uneven sequencing coverage. For example, comparisons between LRR and non-LRR domains do not appear to be sensitive to false positives, as increasing the stringency of the significance thresholds used to identify the codons under selection did not change the overall pattern between LRR and other regions of the gene (data not shown).

Examining the location of positively selected sites on the 3-dimensional structure of *TLR3* (Figure S7B) showed that although our sequence alignment did not cover the region responsible for the interaction with the viral dsRNA ligand, it did cover the region of dimerisation [47]. For *TLR4* (Figure S7B), many positively selected sites occurred near the region involved in ligand binding [48], although many sites outside of this region also showed positive selection.

## Discussion

Previous studies of the evolution of vertebrate TLRs have tested for purifying, balancing or positive selection; the current study is the first comprehensive examination of episodic positive selection, predicted under a pathogen-mediated model of evolution, in avian TLRs. We examined all ten known avian TLRs and found support for claims that the most pervasive selective force operating on TLRs is purifying selection [15], as signified by ω values <1 for all loci (averaging across all sites and all branches of each gene-tree) (Table 1). The ω values we report are of a similar magnitude to a previous study of avian TLRs, based on approximately half as many taxa [17]. Although our alignments target the extracellular LRR regions of each gene (the region most likely to exhibit adaptive responses to co-evolutionary interactions with pathogens [31]), it is not surprising that even these regions have ω values <1 overall, as many residues are highly conserved in this domain to provide the rigid structural framework necessary for PAMP binding [31]. It is more noteworthy that the range of values (0.196–0.551) and their mean (0.390) are much higher than found for most proteins (e.g. [49]–[51]).

Despite pervasive purifying selection, we found evidence of episodic positive selection in the region sequenced for all loci. The proportion of positively selected codons varied across genes (Table 1), but an average of 4.5% of residues showed episodic positive selection (i.e. detected under MEME), consistent with a pathogen-mediated model of evolution: repeated rounds of positive selection, interspersed with purifying selection. The numbers of codons under pervasive positive selection (SLAC and REL results) were similar to those observed in a previous study using fewer taxa [17], but as predicted we found a much larger number of avian TLR codons evolve under episodic selection. These data support the prediction that MEME has greater power to detect sites that experience positive selection in only a subset of lineages, which can be missed by site-based methods [20]. Episodic selection was detected at fewer sites when using a more stringent alpha value (2.6% of codons), but did not change our conclusions regarding the distribution of positively selected codons between LRR and other domains. At alpha = 0.05 the false-positive rates of MEME are generally low (1–2%) for a variety of tree divergence levels [20].

Our results echo findings from different immunogenetic contexts; for example, episodic positive selection was found to play a key role in the evolution of killer cell Ig-like receptor sequences in New World primates [22] and in the evolution of a cluster of disease-response genes within the Poaceae (true grasses) [52]. Avian *TLR4* had the second-highest proportion of positively-selected codons (after *TLR5*, Table 1), concordant with an observation that *TLR4* showed a higher degree of episodic selection than *TLR7* in rats and mice [8]. Comparisons of our results to those from other taxa and genes are only possible when considering pervasive positive selection (as few studies have examined episodic selection). Nevertheless, a large number of *TLR4* residues also appear to evolve under this type of selection, as compared to other TLRs, in both mammals [18], [53] and birds [17]. It is thought that avian and mammalian *TLR4* have similar functions (e.g. [54]), and while the locus is most well-known for its recognition of LPS in gram-negative bacteria [55], it has also been implicated in resilience to other pathogens including fungi, viruses and protozoa [56]–[58]. In a previous study, genotypic variation at *TLR4* (but not at other TLRs) was associated with juvenile survival in a wild population of a threatened bird [24]; *TLR4* also had the highest number of haplotypes (compared to other TLRs) in this bottlenecked population [7]. Variation in this gene was also recently associated with variation in susceptibility to several infectious diseases in chickens [58]. Taken together, these empirical findings support our observation of a role of episodic selection in the evolution of *TLR4* in particular, and suggest that variation at this gene appears to be of evolutionary significance in wild populations. Population-level studies may be particularly useful for revealing the small-scale pressures exerted on variation at this locus.

The lowest proportion of selected sites was observed within the analysed region of *TLR21* (Table 1; Figure 3), although this alignment also contained the fewest taxa. Avian *TLR21* is known to be shared with fish and amphibians [13], and is thought to act as a functional homologue to mammalian *TLR9* [29]. *TLR21* also showed below-average haplotype diversity (number of alleles) in samples of wild populations of house finch and lesser kestrel [17]. These finding may suggest that *TLR21* shows a higher degree of conservation than other avian TLRs.

### Putative functional significance of observations

Several lines of evidence suggest putatively functional consequences of variation in the LRR domains of avian TLRs, specifically the sites identified here as subject to episodic selection. First, for the four loci where our alignments contain codons in both LRR and other domains of the gene, we observed evidence for generally higher ω values, and greater numbers of positively selected sites, in the LRR domains, consistent with predictions based on gene function [31] and a recent observation in mammals [18]. One locus (*TLR2A*) showed a greater degree of positive selection on terminal branches, perhaps indicating a higher degree of species-specific (potentially more recent) positive selection. This pattern may also occur if the positively selected substitutions observed in these loci are beneficial to certain species, but deleterious in other contexts, and thus have a lower chance of being incorporated into long-term evolutionary change.

A second indication of the functional significance of the positively selected sites identified here is the observation that amino acids at positively selected codons were typically more divergent from each other than inferred substitutions. This finding parallels observations in MHC, where profound amino acid substitutions are more likely to occur in the antigen-binding regions [59].

Third, where crystallographic structures were available (*TLR3* and *TLR4*), we see that the vast majority of positively selected sites are concentrated outside of α and β strands of the molecules, in unbound strands that are more free to vary and able to interact with other macromolecules. In addition, several positively selected sites were observed in the putative PAMP binding region of *TLR4* [48] (Figure S7B). This pattern is similar to previous observations in MHC, where codons in the antigen-binding region (exon 2) also experience greater positive selection [60].

### Conclusion

We found that episodic positive selection has played an important role in the evolution of most avian TLRs, consistent with their role in pathogen recognition and a hypothesis of host-pathogen coevolution. Our study also adds to a growing body of evidence implicating *TLR4* as playing a particularly important role in the immunogenetics of wild animals, especially birds. Our results indicate that pathogen-mediated selection pressure may play a role in the evolution of these genes across a variety of taxa. Population-level studies of these processes, such as examination of the association between genotype and disease prevalence/intensity, will likely yield further insight into the role of TLR diversity in natural populations.

## Supporting Information

Table S1 **NCBI accession numbers of Toll-like receptor sequences included in this study. Data are sorted alphabetically by taxon name.**
(DOCX)

Table S2 **Sites identified as being under positive selection using three alternative approaches (SLAC, REL or MEME; see Methods), as compared to sites identified in a previous analysis (** [**17**] **; only SLAC and REL used).**
(DOCX)

Table S3 **Results of** ***t*** **-tests comparing the physicochemical distances of all inferred amino acid substitutions, to distances among amino acid variants observed at positively-selected sites.**
(DOCX)

Table S4 **Results of linear regression examining the effect of domain (“LRR” or “other”) on mean normalised** ***d_N_*** **-** ***d_S_*** **values, for the four TLR loci for which such comparison was possible.**
(DOCX)

Figure S1 **Neighbour-joining trees of 10 TLR loci, used as the basis for evolutionary analyses.** Tip labels are genus names (full species names and Genbank accession numbers provided in Table S1; alignments provided in File S1).
(TIF)

Figure S2 **Alignment of chicken (Genbank protein AAL49971) and human (3FXI_A) partial TLR4 proteins.** The region examined in this study is shown in bold font; positively selected residues (MEME analysis) are shaded grey.
(TIF)

Figure S3 **Alignment of chicken (Genbank protein AAL49971) and mouse (3CIY_A) partial TLR3 proteins.** The region examined in this study is shown in bold font; positively selected residues (MEME analysis) are shaded grey.
(TIF)

Figure S4 **Inferred amino acid substitutions observed across all 10 TLR alignments; intensity of shading correlates with the number of substitutions (range 0–212).**
(TIF)

Figure S5 **Location of positively selected codons (as detected by MEME analysis) on each TLR gene tree.** Codons given refer to positions in the current alignments (File S1); to identify the corresponding position in chicken TLR proteins, refer to Table S2.
(TIF)

Figure S6 **Comparison of mean physicochemical distances between all inferred amino-acid substitutions (open circles) and amino-acid variants observed at positively selected sites (filled circles), at each locus.** Error bars are ±1.96× standard error; asterisks indicate pairs of means that differ at α = 0.05 (full test statistics and sample sizes provided in Table S3).
(TIF)

Figure S7 **Approximate positions of positively-selected residues (MEME analysis; yellow) in the three-dimensional structure of the TLR3 (mouse, MMDB 64341; panel A) and TLR4 (human, MMDB 70004; panel B) homodimer ectodomains (blue and pink) (protein alignments are provided in Figure S2 and S3).** Both molecules are shown from two angles (upper and lower images), and the sequenced region is indicated using a thicker line than the non-sequenced region. Also shown is the association between TLR3 and its dsRNA ligand (green and brown) (in A), and myeloid differentiation factor 2 (MD-2; green and brown), which complexes with TLR4 (in B). Both of these molecular representations are provided as interactive, Cn3D graphics, as Files S2 and S3.
(TIF)

File S1 **Compressed “zip” file containing all ten alignments used in this analysis, in .fas (fasta) format.** Sequences are labelled with Genbank accession numbers and species binom; see also Table S1.
(ZIP)

File S2 **cn3 file for TLR3 structure shown in Figure S7A.** This graphic can be viewed using the NCBI application Cn3D [46].
(CN3)

File S3 **cn3 file for TLR4 structure shown in Figure S7B.** This graphic can be viewed using the NCBI application Cn3D [46].
(CN3)
