# Supplementary material for: Episodic Positive Selection in the Evolution of Avian Toll-Like Receptor Innate Immunity Genes
Source: PLoS One. 2014 Mar 3;9(3):e89632. doi: 10.1371/journal.pone.0089632 (PMC3940441; doi:10.1371/journal.pone.0089632)
Supplement: Table S1 — NCBI accession numbers of Toll-like receptor sequences included in this study. Data are sorted alphabetically by taxon name. (DOCX) [file pone.0089632.s001.docx]

Table S1. NCBI accession numbers of Toll-like receptor sequences included in this study. Data are sorted alphabetically by taxon name.

| Locus | Order | Species | Accession |
| --- | --- | --- | --- |
| *TLR1LA* | Accipitriformes | *Accipiter cooperii* | GU904995 |
|  | Apterygiformes | *Apteryx mantelli* | KF265255 |
|  | Casuariformes | *Dromaius novaehollandiae* | GU904989 |
|  | Falconiformes | *Falco naumanni* | GU904861 |
|  | Galliformes | *Gallus gallus* | NM_001007488 |
|  | Gruiformes | *Porphyrio hochstetteri* | KF265263 |
|  | Passeriformes | *Callaeas wilsoni* | KF265256 |
|  |  | *Carpodacus mexicanus* | GU904709 |
|  |  | *Mohoua ochrocephala* | KF265259 |
|  |  | *Notiomystis cincta* | KF265260 |
|  |  | *Petroica australis rakiura* | JX502626 |
|  |  | *Philesturnus carunculatus* | KF265261 |
|  |  | *Taeniopygia guttata*^*^ | NW_002198637: 1935740‐1938669 |
|  |  | *Xenicus gilviventris* | KF265265 |
|  | Piciformes | *Picoides pubescens* | GU904994 |
|  | Procellariiformes | *Oceanodroma leucorhoa* | GU904993 |
|  | Psittaciformes | *Amazona albifrons* | GU904992 |
|  |  | *Cyanoramphus novaezelandiae* | KF265257 |
|  |  | *Strigops habroptilus* | KF265264 |
| *TLR1LB* | Accipitriformes | *Accipiter cooperii* | GU904946 |
|  | Falconiformes | *Falco naumanni* | GU904872 |
|  | Galliformes | *Gallus gallus* | NM_001081709 |
|  | Gruiformes | *Porphyrio hochstetteri* | KF265273 |
|  | Passeriformes | *Callaeas wilsoni* | KF265266 |
|  |  | *Carpodacus mexicanus* | GU904771 |
|  |  | *Mohoua ochrocephala* | KF265269 |
|  |  | *Notiomystis cincta* | KF265271 |
|  |  | *Petroica australis rakiura* | JX502629 |
|  |  | *Philesturnus carunculatus* | KF265272 |
|  |  | *Taeniopygia guttata*^*^ | NW_002198637: 1925319-1926697 |
|  |  | *Xenicus gilviventris* | KF265274 |
|  | Piciformes | *Picoides pubescens* | GU904949 |
|  | Procellariiformes | *Oceanodroma leucorhoa* | GU904947 |
|  | Psittaciformes | *Amazona albifrons* | GU904948 |
|  |  | *Cyanoramphus novaezelandiae* | KF265267 |
| *TLR2A* | Accipitriformes | *Accipiter cooperii* | GU904953 |
|  | Anseriformes | *Anas platyrhynchos* | FJ477862 |
|  |  | *Anser cygnoides* | JN982474 |
|  | Falconiformes | *Falco naumanni* | GU904887 |
|  | Galliformes | *Gallus gallus* | NM_204278 |
|  |  | *Meleagris gallopavo* | FJ477860 |
|  | Passeriformes | *Carpodacus mexicanus* | GU904791 |
|  |  | *Petroica australis rakiura* | JX502632 |
|  |  | *Taeniopygia guttata*^*^ | NW_002198634: 13604640-13606978 |
|  | Procellariiformes | *Oceanodroma leucorhoa* | GU904954 |
|  | Psittaciformes | *Amazona albifrons* | GU904952 |
| *TLR2B* | Accipitriformes | *Accipiter cooperii* | GU904960 |
|  | Anseriformes | *Anas platyrhynchos* | FJ477862 |
|  | Apterygiformes | *Apteryx mantelli* | KF265276 |
|  | Casuariformes | *Dromaius novaehollandiae* | GU904956 |
|  | Galliformes | *Gallus gallus* | NM_001161650 |
|  |  | *Meleagris gallopavo* | FJ477861 |
|  | Passeriformes | *Notiomystis cincta* | KF265279 |
|  |  | *Petroica australis rakiura* | JX502635 |
|  |  | *Philesturnus carunculatus* | KF265280 |
|  |  | *Taeniopygia guttata*^*^ | NW_002198634: 13607128-13614664 |
|  | Procellariiformes | *Oceanodroma leucorhoa* | GU904959 |
|  | Psittaciformes | *Amazona albifrons* | GU904958 |
|  |  | *Cyanoramphus novaezelandiae* | KF265277 |
|  |  | *Strigops habroptilus* | KF265281 |
| *TLR3* | Accipitriformes | *Accipiter cooperii* | HQ267385 |
|  | Apterygiformes | *Apteryx mantelli* | KF265282 |
|  | Casuariformes | *Dromaius novaehollandiae* | GU904961 |
|  | Columbiformes | *Columba livia* | AB618533 |
|  | Falconiformes | *Falco naumanni* | GU904898 |
|  | Galliformes | *Gallus gallus* | NM_001011691 |
|  | Gruiformes | *Porphyrio hochstetteri* | KF265288 |
|  | Passeriformes | *Callaeas wilsoni* | KF265283 |
|  |  | *Carpodacus mexicanus* | GU904804 |
|  |  | *Mohoua ochrocephala* | KF265285 |
|  |  | *Petroica australis rakiura* | JX502638 |
|  |  | *Philesturnus carunculatus* | KF265287 |
|  |  | *Taeniopygia guttata*^*^ | NW_002198636: 2625536-2631659 |
|  |  | *Xenicus gilviventris* | KF265290 |
|  | Piciformes | *Picoides pubescens* | GU904964 |
|  | Psittaciformes | *Cyanoramphus novaezelandiae* | KF265284 |
|  |  | *Strigops habroptilus* | KF265289 |
| *TLR4* | Accipitriformes | *Accipiter cooperii* | GU904970 |
|  | Anseriformes | *Anas platyrhynchos* | JQ839148 |
|  | Casuariformes | *Dromaius novaehollandiae* | GU904966 |
|  | Falconiformes | *Falco naumanni* | GU904900 |
|  | Galliformes | *Gallus gallus* | NM_001030693 |
|  |  | *Perdix perdix* | JQ713172 |
|  | Gruiformes | *Porphyrio hochstetteri* | KF265299 |
|  | Passeriformes | *Carpodacus mexicanus* | GU904813 |
|  |  | *Mohoua ochrocephala* | KF265293 |
|  |  | *Notiomystis cincta* | KF265295 |
|  |  | *Petroica australis rakiura* | JX502640 |
|  |  | *Philesturnus carunculatus* | KF265297 |
|  |  | *Taeniopygia guttata* | NM_001142454 |
|  |  | *Xenicus gilviventris* | KF265301 |
|  | Piciformes | *Picoides pubescens* | GU904971 |
|  | Procellariiformes | *Oceanodroma leucorhoa* | GU904969 |
|  | Psittaciformes | *Cyanoramphus novaezelandiae* | KF265292 |
|  |  | *Strigops habroptilus* | KF265300 |
| *TLR5* | Accipitriformes | *Accipiter cooperii* | GU904974 |
|  | Anseriformes | *Anser anser* | JX096947 |
|  | Apterygiformes | *Apteryx mantelli* | KF265303 |
|  | Casuariformes | *Dromaius novaehollandiae* | GU904972 |
|  | Falconiformes | *Falco naumanni* | GU904907 |
|  | Galliformes | *Gallus gallus* | NM_001024586 |
|  |  | *Meleagris gallopavo* | HQ436463 |
|  |  | *Perdix perdix* | JQ713180 |
|  | Gruiformes | *Porphyrio hochstetteri* | KF265312 |
|  | Passeriformes | *Callaeas wilsoni* | KF265304 |
|  |  | *Mohoua ochrocephala* | KF265308 |
|  |  | *Notiomystis cincta* | KF265310 |
|  |  | *Petroica australis rakiura* | JX502646 |
|  |  | *Philesturnus carunculatus* | KF265311 |
|  |  | *Taeniopygia guttata*^*^ | NW_002198506: 243510-246064 |
|  | Piciformes | *Picoides pubescens* | GU904976 |
|  | Procellariiformes | *Oceanodroma leucorhoa* | GU904975 |
|  | Psittaciformes | *Cyanoramphus novaezelandiae* | KF265307 |
|  |  | *Strigops habroptilus* | KF265313 |
| *TLR7* | Accipitriformes | *Accipiter cooperii* | GU904982 |
|  | Anseriformes | *Anser cygnoides* | JQ910168 |
|  | Apterygiformes | *Apteryx mantelli* | KF265315 |
|  | Casuariformes | *Dromaius novaehollandiae* | GU904977 |
|  | Falconiformes | *Falco naumanni* | GU904923 |
|  | Galliformes | *Coturnix japonica* | AB553582 |
|  |  | *Gallus gallus* | NM_001011688 |
|  |  | *Meleagris gallopavo* | XM_003203086 |
|  |  | *Perdix perdix* | JQ713178 |
|  | Gruiformes | *Porphyrio hochstetteri* | KF265323 |
|  | Passeriformes | *Callaeas wilsoni* | KF265316 |
|  |  | *Carpodacus mexicanus* | GU904828 |
|  |  | *Mohoua ochrocephala* | KF265319 |
|  |  | *Notiomystis cincta* | KF265320 |
|  |  | *Petroica australis rakiura* | JX502658 |
|  |  | *Philesturnus carunculatus* | KF265322 |
|  |  | *Taeniopygia guttata*^*^ | NW_002197669: 14707528-14718808 |
|  |  | *Xenicus gilviventris* | KF265325 |
|  | Piciformes | *Picoides pubescens* | GU904983 |
|  | Procellariiformes | *Oceanodroma leucorhoa* | GU904981 |
|  | Psittaciformes | *Amazona albifrons* | GU904980 |
|  |  | *Cyanoramphus novaezelandiae* | KF265318 |
|  |  | *Strigops habroptilus* | KF265324 |
| *TLR15* | Anseriformes | *Anser anser* | JQ014619 |
|  | Falconiformes | *Falco naumanni* | GU904927 |
|  | Galliformes | *Coturnix coturnix* | HM773176 |
|  |  | *Gallus gallus* | NM_001037835 |
|  |  | *Meleagris gallopavo* | HQ456924 |
|  | Gruiformes | *Porphyrio hochstetteri* | KF265333 |
|  | Passeriformes | *Callaeas wilsoni* | KF265326 |
|  |  | *Carpodacus mexicanus* | GU904843 |
|  |  | *Mohoua ochrocephala* | KF265328 |
|  |  | *Notiomystis cincta* | KF265330 |
|  |  | *Petroica australis rakiura* | JX502650 |
|  |  | *Philesturnus carunculatus* | KF265332 |
|  |  | *Taeniopygia guttata*^*^ | NW_002198506: 20536896-20539486 |
|  | Procellariiformes | *Oceanodroma leucorhoa* | GU904986 |
| *TLR21* | Falconiformes | *Falco naumanni* | GU904941 |
|  | Galliformes | *Gallus gallus* | NM_001030558 |
|  | Gruiformes | *Porphyrio hochstetteri* | KF265339 |
|  | Passeriformes | *Callaeas wilsoni* | KF265335 |
|  |  | *Carpodacus mexicanus* | GU904859 |
|  |  | *Mohoua ochrocephala* | KF265336 |
|  |  | *Notiomystis cincta* | KF265337 |
|  |  | *Petroica australis rakiura* | JX502653 |
|  |  | *Taeniopygia guttata*^*^ | NW_002218839:  35719-38419 |

^*^ zebra finch genomic locations as identified by Alcaide & Edwards (2011)
