# Supplementary material for: Episodic Positive Selection in the Evolution of Avian Toll-Like Receptor Innate Immunity Genes
Source: PLoS One. 2014 Mar 3;9(3):e89632. doi: 10.1371/journal.pone.0089632 (PMC3940441; doi:10.1371/journal.pone.0089632)
Supplement: Table S2 — Sites identified as being under positive selection using three alternative approaches (SLAC, REL or MEME; see Methods), as compared to sites identified in a previous analysis ( [17] ; only SLAC and REL used). (DOCX) [file pone.0089632.s002.docx]

**Table S2.** Sites identified as being under positive selection using three alternative approaches (SLAC, REL or MEME; see Methods), as compared to sites identified in a previous analysis (Alcaide and Edwards 2011; only SLAC and REL used).

| Gene | Position^*^ | |  | Method | | Amino acids^†^ |
| --- | --- | --- | --- | --- | --- | --- |
|  | Alignment | Chicken |  | Current study | Previous study |  |
| *TLR1LA* | 12 | 238 |  |  | SLAC | A E K L M T |
|  | 63 | 289 |  | MEME |  | L P |
|  | 67 | 293 |  | REL, MEME |  | A D E K R |
|  | 71 | 297 |  | MEME |  | F I L |
|  | 75 | 301 |  | REL, MEME | REL | A L M Q V |
|  | 120 | 346 |  | MEME |  | A H Y |
|  | 139 | 365 |  | MEME |  | I L |
|  | 158 | 384 |  | REL, MEME |  | A M R T V |
|  | 162 | 388 |  | SLAC, REL, MEME |  | F I L S |
|  | 212 | 438 |  | MEME |  | A M S T |
|  | 227 | 453 |  | MEME |  | A E L S |
|  | 234 | 460 |  | REL, MEME |  | A E K N Q R |
|  | 288 | 514 |  | MEME |  | I V |
|  | 324 | 550 |  | MEME |  | E G L V |
| *TLR1LB* | 12 | 148 |  | MEME |  | A E I K N T V |
|  | 15 | 151 |  | MEME |  | A D K R Y |
|  | 28 | 164 |  | MEME |  | L M V |
|  | 32 | 168 |  | MEME | REL | A I L Q V |
|  | 39 | 175 |  | MEME |  | I S T |
|  | 71 | 207 |  | MEME |  | D K N R S |
|  | 96 | 232 |  | MEME |  | D I L M T |
|  | 150 | 286 |  | MEME |  | A M S T |
|  | 165 | 301 |  | MEME |  | A E L S |
|  | 172 | 308 |  | MEME |  | A E K N Q R |
|  | 175 | 311 |  | MEME |  | D G N S |
|  | 226 | 362 |  | MEME |  | I V |
|  | 262 | 398 |  | SLAC, MEME |  | E G L V |
| *TLR2A* | 4 | 235 |  | MEME |  | I T V |
|  | 12 | 243 |  | MEME |  | A K R |
|  | 19 | 250 |  | MEME |  | H K S T |
|  | 33 | 264 |  | MEME |  | L M |
|  | 39 | 270 |  | MEME |  | F S |
|  | 44 | 275 |  | MEME |  | T Y |
|  | 49 | 280 |  | REL |  | A I L M |
|  | 61 | 292 |  | MEME |  | I L V |
|  | 73 | 304 |  | REL, MEME | SLAC | A D E I M N S |
|  | 77 | 308 |  | REL, MEME |  | E K T |
|  | 78 | 309 |  | MEME |  | A E L |
|  | 80 | 311 |  | MEME |  | A K |
|  | 89 | 320 |  | MEME |  | A S T |
|  | 90 | 321 |  | MEME |  | I L V |
|  | 92 | 323 |  | MEME |  | I L V |
|  | 161 | 392 |  | REL, MEME |  | E K N Q R |
|  | 162 | 393 |  | REL, MEME |  | D G H N Y |
|  | 187 | 418 |  | REL, MEME |  | I T V |
|  | 231 | 462 |  | MEME |  | A G R T |
|  | 256 | 487 |  | MEME |  | F L N Y |
|  | 274 | 505 |  | MEME |  | A M T |
|  | 275 | 506 |  | MEME |  | A D G P S |
|  | 330 | 561 |  | MEME |  | A G S T |
|  | 356 | 587 |  | MEME |  | I V |
|  | 372 | 603 |  | MEME |  | I V |
| *TLR2B* | 2 | 237 |  | MEME |  | A E Q T |
|  | 37 | 272 |  | MEME |  | A I L |
|  | 58 | 293 |  |  | SLAC | H K L Q R |
|  | 60 | 295 |  | MEME | SLAC | D E K Q S Y |
|  | 61 | 296 |  |  | SLAC | I K L M T |
|  | 62 | 297 |  | MEME |  | E H P Q |
|  | 65 | 300 |  | SLAC, MEME |  | A E I V |
|  | 73 | 308 |  | MEME |  | I V |
|  | 94 | 329 |  | MEME |  | E K L Q V |
|  | 120 | 355 |  |  | SLAC | H K N Q R |
|  | 137 | 372 |  | MEME |  | L Q |
|  | 215 | 450 |  | MEME |  | A G N R T |
|  | 286 | 521 |  | MEME |  | H K Q R |
|  | 356 | 591 |  | MEME |  | I V |
|  | 363 | 598 |  | MEME |  | A F L V |
| *TLR3* | -37 | 210 |  |  | SLAC |  |
|  | -13 | 234 |  |  | SLAC |  |
|  | 6 | 253 |  |  | SLAC | L |
|  | 17 | 264 |  | REL, MEME |  | A G N S |
|  | 33 | 280 |  |  | SLAC | N Y |
|  | 43 | 290 |  | MEME |  | A E |
|  | 59 | 306 |  | MEME |  | A E K |
|  | 60 | 307 |  | REL, MEME |  | D H I L N R |
|  | 65 | 312 |  | REL, |  | H N R T |
|  | 87 | 334 |  | REL, MEME |  | I N S T |
|  | 159 | 406 |  |  | SLAC | T |
|  | 192 | 439 |  | REL, MEME |  | E K R T |
|  | 202 | 449 |  | MEME |  | I L V |
|  | 214 | 461 |  | SLAC, REL, MEME |  | A C D I S T |
|  | 216 | 463 |  | SLAC, REL, MEME |  | K Q R |
|  | 250 | 497 |  | REL, |  | K Q R |
|  | 306 | 553 |  | MEME |  | P Q R T |
|  | 329 | 576 |  |  | SLAC | L |
|  | 354 | 601 |  | SLAC, REL, MEME |  | A N T V |
| *TLR4* | 18 | 221 |  | MEME |  | C H S Y |
|  | 43 | 246 |  | MEME |  | A D M T V |
|  | 47 | 250 |  | MEME |  | A S |
|  | 67 | 270 |  | REL, MEME |  | I K N Q R S |
|  | 68 | 271 |  | REL, MEME |  | E G K L Q R W |
|  | 71 | 274 |  |  | SLAC | G K Q R T V |
|  | 98 | 301 |  | REL, |  | D E G K R |
|  | 99 | 302 |  | MEME |  | D G N S Y |
|  | 120 | 323 |  | MEME |  | G N Q |
|  | 130 | 333 |  | REL, |  | F G L S W |
|  | 142 | 345 |  | MEME |  | D G H K R |
|  | 149 | 352 |  | SLAC, REL, MEME | SLAC, REL | E K L M Q T V |
|  | 162 | 365 |  | MEME |  | I L M |
|  | 167 | 370 |  | REL, MEME |  | D G H N R S Y |
|  | 172 | 375 |  | REL, |  | E K N Q R |
|  | 184 | 387 |  | REL, |  | I L M V |
|  | 194 | 397 |  | REL, MEME | SLAC | G K L P S T Y |
|  | 195 | 398 |  | REL, MEME |  | G H L R S T |
|  | 199 | 402 |  | MEME |  | L P R T |
|  | 203 | 406 |  | MEME | SLAC | D G K N R |
|  | 232 | 435 |  | MEME |  | T Y |
|  | 244 | 447 |  | MEME |  | G K |
|  | 245 | 448 |  | MEME |  | N S T Y |
|  | 269 | 472 |  | MEME |  | G S |
| *TLR5* | 8 | 209 |  | REL, MEME |  | E H L N Q S T |
|  | 11 | 212 |  | MEME |  | H K M |
|  | 13 | 214 |  | MEME |  | D E S |
|  | 14 | 215 |  | MEME |  | D E K L |
|  | 36 | 237 |  | REL, |  | D E H K N Q R S |
|  | 43 | 244 |  |  | SLAC | K Q R S T |
|  | 57 | 258 |  |  | SLAC | D G N S T |
|  | 60 | 261 |  | REL, MEME | SLAC | I K M N S T V |
|  | 63 | 264 |  | SLAC, REL, MEME | SLAC | F H L P S T |
|  | 64 | 265 |  | MEME |  | H T |
|  | 75 | 276 |  | MEME |  | F I L |
|  | 77 | 278 |  | MEME |  | G N |
|  | 80 | 281 |  | SLAC, REL, |  | D E K N Q S T Y |
|  | 81 | 282 |  | MEME |  | D L N S |
|  | 92 | 293 |  | SLAC, REL, |  | H K N R S |
|  | 98 | 299 |  | SLAC, REL, MEME |  | D H N Q R |
|  | 105 | 306 |  | MEME |  | K N Q |
|  | 108 | 309 |  |  | SLAC | I T V |
|  | 121 | 322 |  | MEME |  | C F S V Y |
|  | 130 | 331 |  | MEME | SLAC | E K R |
|  | 131 | 332 |  | MEME |  | G H K Q R |
|  | 134 | 335 |  | MEME |  | F W |
|  | 158 | 359 |  | MEME |  | D E T |
|  | 177 | 378 |  | MEME |  | A D E G Q |
|  | 183 | 384 |  | MEME |  | K N Q R S |
|  | 197 | 398 |  | MEME |  | I L V |
|  | 208 | 409 |  | SLAC, MEME |  | F I S T Y |
|  | 221 | 422 |  |  | SLAC | D G H N S Y |
|  | 223 | 424 |  | MEME |  | A E K T |
|  | 254 | 455 |  |  | SLAC | I L |
|  | 265 | 466 |  | MEME | SLAC | E K V |
|  | 270 | 471 |  | MEME | SLAC | A I T V |
|  | 300 | 501 |  | MEME | SLAC | F Q |
|  | 301 | 502 |  | REL, MEME | SLAC | G H K R V |
|  | 303 | 504 |  | MEME |  | G L |
|  | 304 | 505 |  | MEME |  | A L P S T |
|  | 307 | 508 |  | MEME |  | Q R |
|  | 332 | 533 |  | SLAC, MEME |  | G I K R S |
|  | 347 | 548 |  | MEME |  | I L V |
|  | 353 | 554 |  | MEME |  | A I K R T |
|  | 360 | 561 |  |  | SLAC | N |
|  | 361 | 562 |  | MEME |  | M Q |
|  | 369 | 570 |  | MEME |  | I L V |
| *TLR7* | 51 | 257 |  | REL, MEME |  | H L P Y |
|  | 85 | 291 |  | MEME |  | A K M R |
|  | 95 | 301 |  | MEME |  | I T V |
|  | 104 | 310 |  | MEME |  | H Q R |
|  | 114 | 320 |  | REL, MEME |  | I L T |
|  | **119** | 325 |  | MEME |  | D E K N Q T V |
|  | 139 | 345 |  | REL, MEME |  | F H L |
|  | 160 | 366 |  | MEME |  | F S T Y |
|  | 189 | 395 |  | MEME |  | E G Q R |
|  | 221 | 427 |  | MEME |  | F L |
|  | 226 | 432 |  | MEME |  | F I L V |
|  | 238 | 444 |  | MEME |  | G S |
|  | 284 | 490 |  | MEME |  | A V Y |
| *TLR15* | 12 | 128 |  |  | SLAC | I K L S |
|  | 26 | 142 |  | MEME | SLAC | H L N Y |
|  | 43 | 159 |  | MEME |  | E G L |
|  | 47 | 162 |  | REL, MEME |  | A C H P R |
|  | 53 | 168 |  |  | SLAC | D L N V |
|  | 55 | 170 |  | MEME |  | G P Q |
|  | 57 | 172 |  | MEME | SLAC | G L T V |
|  | 76 | 185 |  |  | SLAC | A K T V |
|  | 79 | 188 |  | MEME |  | L P S |
|  | 94 | 197 |  | MEME |  | F L R V |
|  | 107 | 210 |  |  | SLAC | G H K N R Y |
|  | 117 | 220 |  | MEME |  | A E L |
|  | 130 | 233 |  | SLAC, REL, MEME | SLAC | A G K N R S T |
|  | 150 | 253 |  | REL, MEME |  | A I M S T |
|  | 154 | 257 |  | MEME |  | P Y |
|  | 180 | 283 |  | SLAC, REL, MEME | SLAC | A E H K N R S |
|  | 183 | 286 |  | MEME |  | D F L S |
|  | 202 | 305 |  | MEME |  | I L M V |
|  | 220 | 323 |  | MEME |  | Q R T V |
|  | 255 | 358 |  | MEME |  | E P T |
|  | 263 | 364 |  | MEME |  | F T |
|  | 393 | 494 |  | REL, MEME |  | D K N R S |
|  | 410 | 511 |  | MEME |  | H R |
| *TLR21* | 8 | 446 |  | MEME |  | E H |
|  | 32 | 470 |  | MEME |  | D T |
|  | 120 | 558 |  | MEME |  | E L P |

^*^ Position refers to the current alignments, and the corresponding position in chicken TLR protein sequences (accession numbers provided in Methods).

^†^ IUPAC amino-acid codes for amino acid variants observed in the current study; sites that were variable within a species (i.e. the sequence was heterozygous) were ignored.
