# Supplementary material for: Episodic Positive Selection in the Evolution of Avian Toll-Like Receptor Innate Immunity Genes
Source: PLoS One. 2014 Mar 3;9(3):e89632. doi: 10.1371/journal.pone.0089632 (PMC3940441; doi:10.1371/journal.pone.0089632)
Supplement: Table S3 — Results of t -tests comparing the physicochemical distances of all inferred amino acid substitutions, to distances among amino acid variants observed at positively-selected sites. (DOCX) [file pone.0089632.s003.docx]

**Table S3.** Results of *t*-tests comparing the physicochemical distances of all inferred amino acid substitutions, with distances among amino acid variants observed at positively-selected sites.

| Locus | *N*_all_^*^ | *N*_pos_^*^ | Mean # aa^†^ | *t*-statistic^‡^ | df | *P*-value^§^ | |
| --- | --- | --- | --- | --- | --- | --- | --- |
| *TLR1LA* | 536 | 78 | 3.77 | 2.71 | 101.0 | 0.0079 | * |
| *TLR1LB* | 450 | 107 | 4.38 | 4.90 | 156.2 | < 0.0001 | * |
| *TLR2A* | 402 | 117 | 3.36 | 3.26 | 167.6 | 0.0013 | * |
| *TLR2B* | 452 | 71 | 3.75 | 3.71 | 91.9 | 0.0004 | * |
| *TLR3* | 287 | 79 | 3.85 | 3.63 | 118.4 | 0.0004 | * |
| *TLR4* | 481 | 218 | 4.55 | 6.69 | 394.2 | < 0.0001 | * |
| *TLR5* | 602 | 284 | 3.85 | 4.09 | 554.1 | < 0.0001 | * |
| *TLR7* | 367 | 68 | 3.54 | 1.10 | 99.7 | 0.2752 |  |
| *TLR15* | 616 | 126 | 3.89 | 4.67 | 168.5 | < 0.0001 | * |
| *TLR21* | 248 | 5 | 2.33 | 1.58 | 4.2 | 0.1871 |  |
| Overall | 4,441 | 1,157 | 3.91^¶^ | 12.29 | 1,723.3 | < 0.0001 | * |

^*^ Sample sizes are the number of pairwise comparisons between amino acids. For “all”, the data are the physicochemical distances of all inferred substitutions (Figure S2). For “pos”, we calculated the distances among all observed amino acids at each positively selected site (Table S2).

^†^ mean number of amino acid variants observed at positively selected sites

^‡^ Summary statistics for each locus are presented in Figure S2.

^§^ 2-tailed test, *H*_0_: *x̅*_1_ = *x̅*_2_; * = statistically significant deviation from *H*_0_ at α = 0.05.

^¶^ Overall value is taken by first averaging the amino acid distances among all variants at a site, then taking the mean across sites.
