# Supplementary material for: Episodic Positive Selection in the Evolution of Avian Toll-Like Receptor Innate Immunity Genes
Source: PLoS One. 2014 Mar 3;9(3):e89632. doi: 10.1371/journal.pone.0089632 (PMC3940441; doi:10.1371/journal.pone.0089632)
Supplement: Table S4 — Results of linear regression examining the effect of domain (“LRR” or “other”) on mean normalised dN - dS values, for the four TLR loci for which such comparison was possible. (DOCX) [file pone.0089632.s004.docx]

**Table S4.** Results of linear regression examining the effect of domain (“LRR” or “other”) on mean normalised *d_N_*- *d_S_* values, for the four TLR loci for which such comparison was possible.

| Locus^*^ | *N*_LRR_ | *N*_other_ | β (SE_β_)^†^ | *P*-value^‡^ | |
| --- | --- | --- | --- | --- | --- |
| *TLR1LA* | 324 | 63 | -0.343 (0.228) | 0.1337 |  |
| *TLR1LB* | 262 | 54 | -0.322 (0.255) | 0.2079 |  |
| *TLR2A* | 325 | 88 | -0.576 (0.275) | 0.0371 | * |
| *TLR2B* | 309 | 88 | -0.225 (0.186) | 0.2266 |  |

^*^ see Figure 4a for graphical representation of these comparisons.

^†^ effect size and its standard error.

^‡^ 2-tailed test, *H*_0_: β = 0; * = statistically significant deviation from *H*_0_ at α = 0.05.
