# Supplementary figures and images for: Episodic Positive Selection in the Evolution of Avian Toll-Like Receptor Innate Immunity Genes
Source: PLoS One. 2014 Mar 3;9(3):e89632. doi: 10.1371/journal.pone.0089632 (PMC3940441; doi:10.1371/journal.pone.0089632)

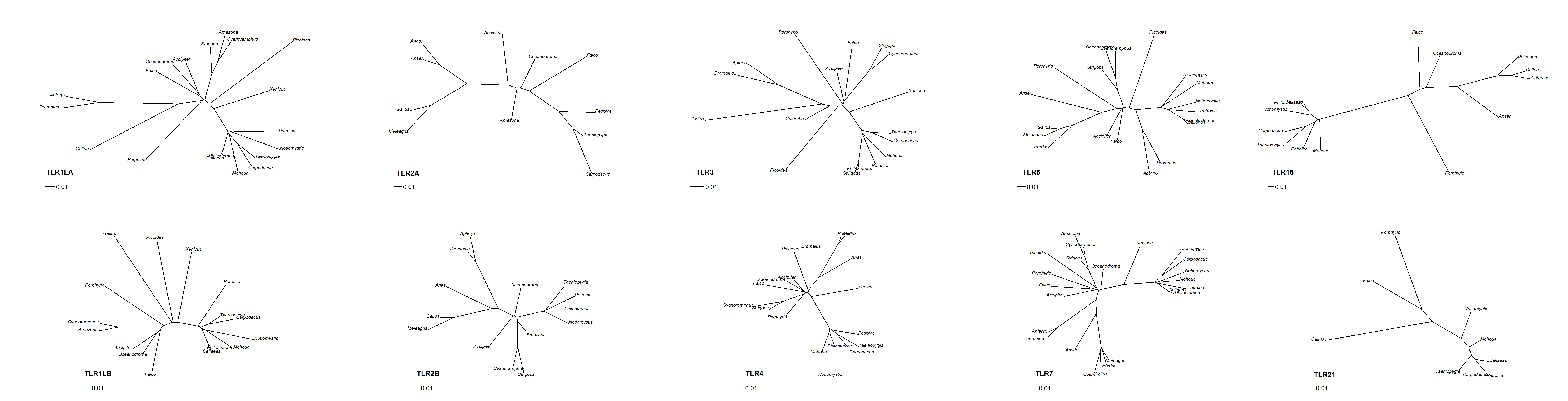

Supplement: Figure S1 — Neighbour-joining trees of 10 TLR loci, used as the basis for evolutionary analyses. Tip labels are genus names (full species names and Genbank accession numbers provided in Table S1; alignments provided in File S1). (TIF) [file pone.0089632.s005.tif]

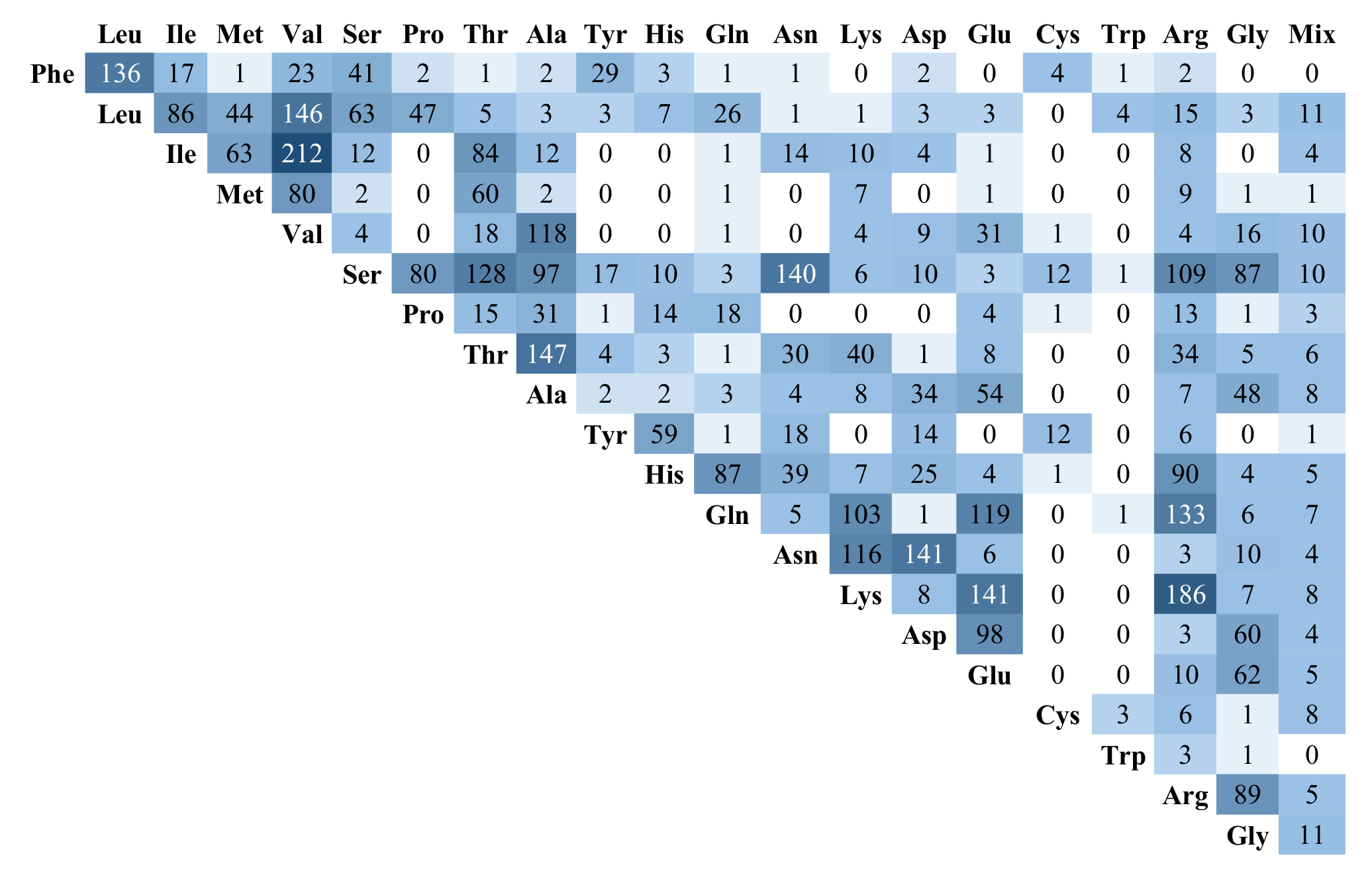

Supplement: Figure S4 — Inferred amino acid substitutions observed across all 10 TLR alignments; intensity of shading correlates with the number of substitutions (range 0–212). (TIF) [file pone.0089632.s008.tif]

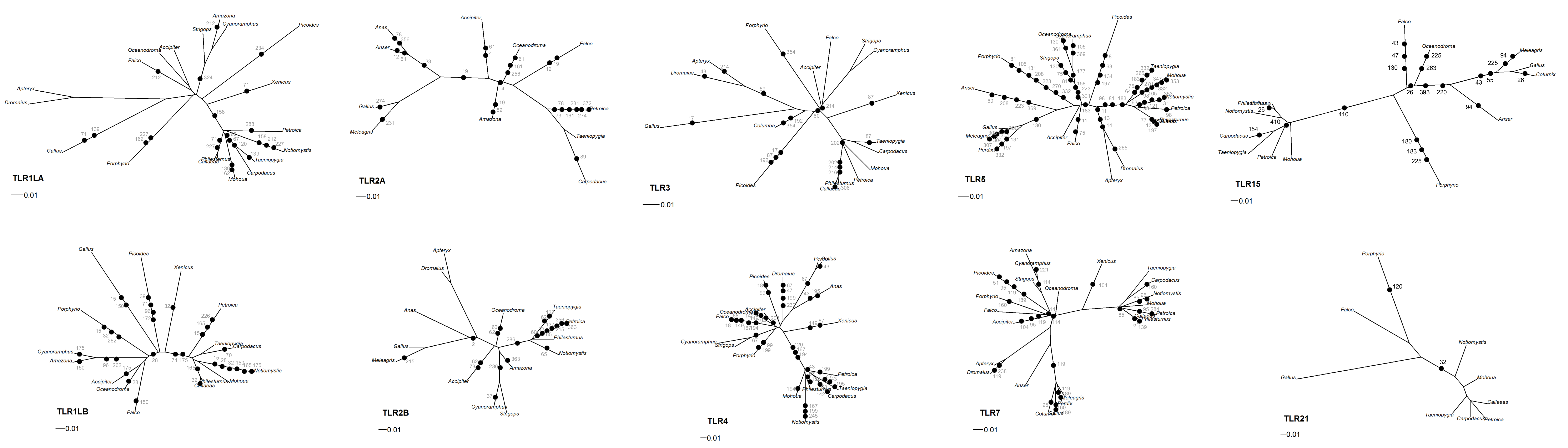

Supplement: Figure S5 — Location of positively selected codons (as detected by MEME analysis) on each TLR gene tree. Codons given refer to positions in the current alignments (File S1); to identify the corresponding position in chicken TLR proteins, refer to Table S2. (TIF) [file pone.0089632.s009.tif]

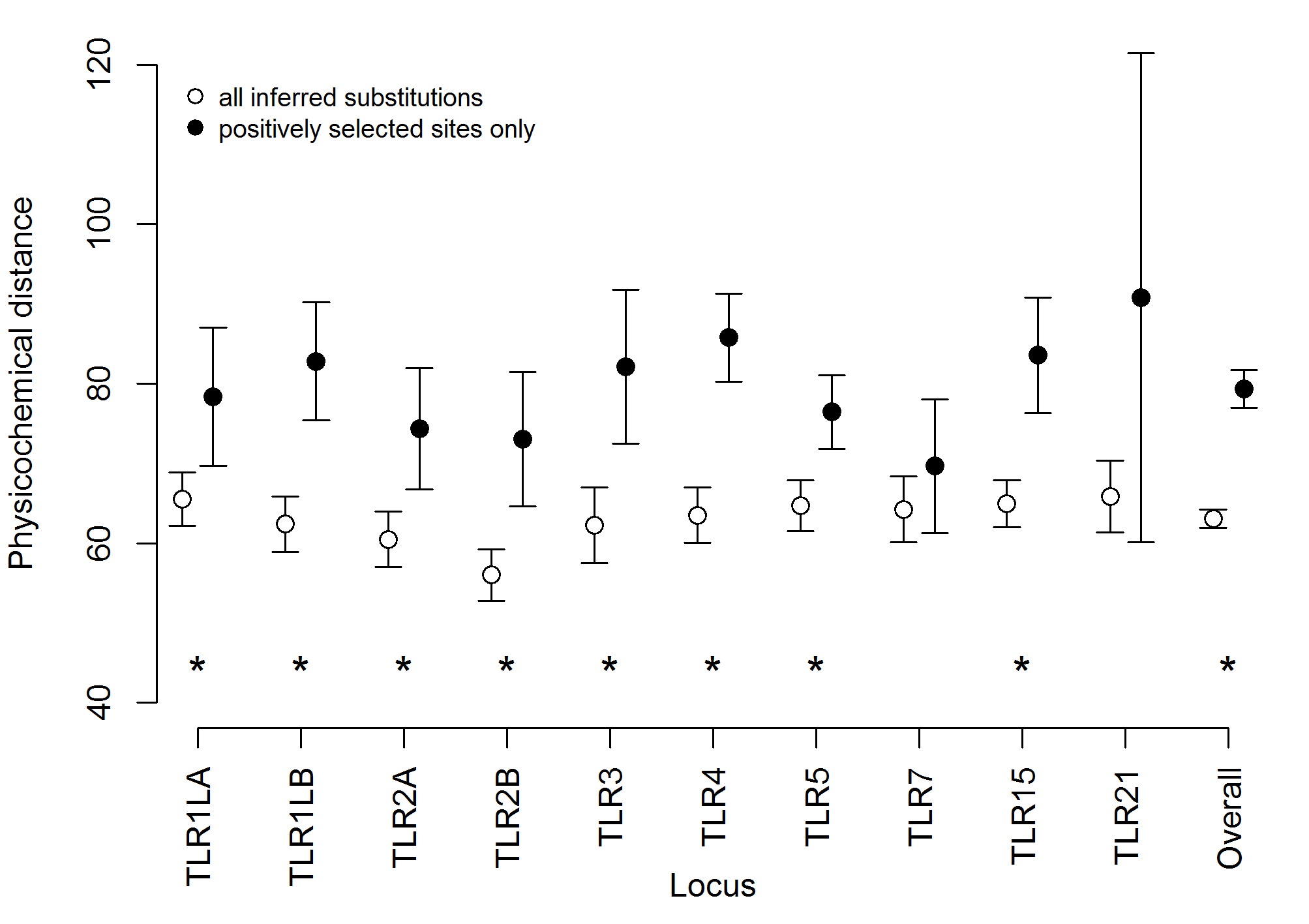

Supplement: Figure S6 — Comparison of mean physicochemical distances between all inferred amino-acid substitutions (open circles) and amino-acid variants observed at positively selected sites (filled circles), at each locus. Error bars are ±1.96× standard error; asterisks indicate pairs of means that differ at α = 0.05 (full test statistics and sample sizes provided in Table S3). (TIF) [file pone.0089632.s010.tif]

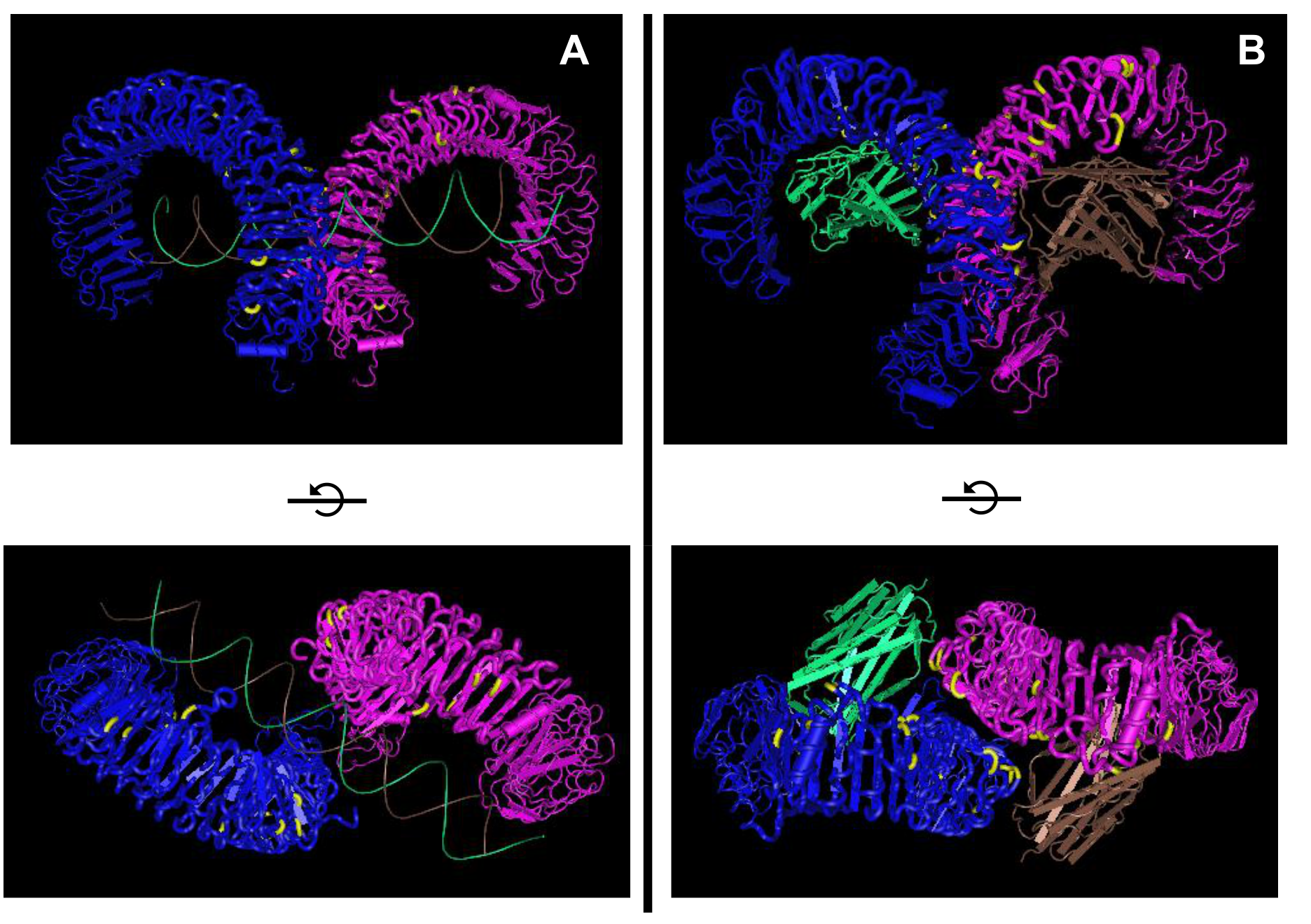

Supplement: Figure S7 — Approximate positions of positively-selected residues (MEME analysis; yellow) in the three-dimensional structure of the TLR3 (mouse, MMDB 64341; panel A) and TLR4 (human, MMDB 70004; panel B) homodimer ectodomains (blue and pink) (protein alignments are provided in Figure S2 and S3). Both molecules are shown from two angles (upper and lower images), and the sequenced region is indicated using a thicker line than the non-sequenced region. Also shown is the association between TLR3 and its dsRNA ligand (green and brown) (in A), and myeloid differentiation factor 2 (MD-2; green and brown), which complexes with TLR4 (in B). Both of these molecular representations are provided as interactive, Cn3D graphics, as Files S2 and S3. (TIF) [file pone.0089632.s011.tif]
